# Computational prediction of the effect of amino acid changes on the binding affinity between SARS-CoV-2 spike RBD and human ACE2

**DOI:** 10.1073/pnas.2106480118

**Published:** 2021-09-29

**Authors:** Chen Chen, Veda Sheersh Boorla, Deepro Banerjee, Ratul Chowdhury, Victoria S. Cavener, Ruth H. Nissly, Abhinay Gontu, Nina R. Boyle, Kurt Vandegrift, Meera Surendran Nair, Suresh V. Kuchipudi, Costas D. Maranas

**Affiliations:** ^a^Department of Chemical Engineering, The Pennsylvania State University, University Park, PA 16802;; ^b^The Bioinformatics and Genomics Program, Huck Institutes of the Life Sciences, The Pennsylvania State University, University Park, PA 16802;; ^c^Department of Veterinary and Biomedical Sciences, The Pennsylvania State University, University Park, PA 16802;; ^d^Animal Diagnostic Laboratory, Department of Veterinary and Biomedical Sciences, The Pennsylvania State University, University Park, PA 16802;; ^e^Center for Infectious Disease Dynamics, The Pennsylvania State University, University Park, PA 16802

**Keywords:** SARS-CoV-2, human ACE2, binding affinity, MM-GBSA, neural network

## Abstract

The association of the receptor binding domain (RBD) of severe acute respiratory syndrome coronavirus 2 (SARS-CoV-2) spike protein with human angiotensin-converting enzyme 2 (hACE2) represents the first required step for cellular entry. SARS-CoV-2 has continued to evolve with the emergence of several novel variants, and amino acid changes in the RBD have been implicated with increased fitness and potential for immune evasion. Reliably predicting the effect of amino acid changes on the ability of the RBD to interact more strongly with the hACE2 can help assess the implications for public health and the potential for spillover and adaptation into other animals. Here, we introduce a two-step framework that first relies on 48 independent 4-ns molecular dynamics (MD) trajectories of RBD−hACE2 variants to collect binding energy terms decomposed into Coulombic, covalent, van der Waals, lipophilic, generalized Born solvation, hydrogen bonding, π−π packing, and self-contact correction terms. The second step implements a neural network to classify and quantitatively predict binding affinity changes using the decomposed energy terms as descriptors. The computational base achieves a validation accuracy of 82.8% for classifying single–amino acid substitution variants of the RBD as worsening or improving binding affinity for hACE2 and a correlation coefficient of 0.73 between predicted and experimentally calculated changes in binding affinities. Both metrics are calculated using a fivefold cross-validation test. Our method thus sets up a framework for screening binding affinity changes caused by unknown single– and multiple–amino acid changes offering a valuable tool to predict host adaptation of SARS-CoV-2 variants toward tighter hACE2 binding.

## Significance

SARS-CoV-2 infection proceeds through the binding of viral surface spike protein to the human ACE2 protein. The global spread of the infection has led to the emergence of fitter and more transmissible variants with increased adaptation both in human and nonhuman hosts. Molecular simulations of the binding event between the spike and ACE2 proteins offer a route to assess potential increase or decrease in infectivity by measuring the change in binding strength. We trained a neural network model that accurately maps simulated binding energies to experimental changes in binding strength upon amino acid changes in the spike protein. This computational workflow can be used to a priori assess currently circulating and prospectively future viral variants for their affinity for hACE2.

The ongoing COVID-19 pandemic caused by severe acute respiratory syndrome coronavirus 2 (SARS-CoV-2) continues to be a major global challenge to public health and has caused unprecedented losses to the global economy (1) and ecology (2). Multiple vaccines have received emergency use authorization (Pfizer, Moderna, and J&J), and additional vaccines are yet to receive authorization (AstraZeneca and Novavax) in the United States. However, several new variants of the wild-type (WT) virus (i.e., isolate Wuhan-Hu-1, GenBank ID code MN908947) have emerged in United Kingdom (3) (B.1.1.7 or α), South Africa (4) (B.1.351 or β), Brazil (5) (P.1 or γ), California (6) (B.1.429), New York (7) (B.1.526), and, more recently, India (8) (B.1.617.2 or δ) and Peru (9) (C.37 or λ), with increasing prevalence worldwide. The emergence of novel variants is expected to continue as the virus faces increasing immune pressure due to an expanding proportion of the host population being vaccinated and/or getting immune from natural infection. These variants include one or more nonsynonymous mutations leading to amino acid changes in the spike protein. The amino acid changes may confer fitness advantages and increased infectivity through a variety of mechanisms. Increased binding affinity of the receptor binding domain (RBD) of the spike protein with the human angiotensin-converting enzyme-2 (hACE2) receptor (10) is one such mechanism, although changes in the conformational dynamics of the spike protein (11) have also been implicated. Although recent reports suggest that the current vaccines can still effectively protect people from SARS-CoV-2 variants (12–14), plasma from recipients of Moderna (mRNA-1273) or Pfizer-BioNTech (BNT162b2) vaccines is shown to be less effective in neutralizing SARS-CoV-2 variants encoding E484K or N501Y or the K417N+E484K+N501Y (15) amino acid changes. In addition, a decrease in neutralizing titers against the B.1.351(β) but not the B.1.1.7(β) UK variant with plasma from mRNA-1273 vaccinated humans and nonhuman primates has been observed (16). Hence, continued surveillance and methods to accurately predict affinity gains of the RBD−hACE2 binding event due to amino acid changes in the RBD remain important.

SARS-CoV-2 is an enveloped virus with a single-stranded RNA genome of ∼30-kb size (17). The mutation rates of RNA viruses upon replication are generally higher than DNA viruses, which could be as high as 10^−4^ to 10^−3^ per nucleotide incorporated (18). SARS-CoV-2 has a mutation rate of, on average, 7.23 mutations per sample (19, 20), which is significantly lower than that of HIV and influenza A viruses (20, 21). The simultaneous incorporation of multiple (i.e., refs. 15–20) amino acid changes in a few emerging strains such as ΔFVI (Danish mink), B.1.1.7 (α) (United Kingdom), B.1.1.54 (South Africa) is a cause of concern as it suggests further adaptation of the virus and fitness gains in humans and other animals (22–24). Several of these variants involve amino acid changes in the spike protein suspected to increase transmissibility (25), alter infectivity (26, 27), and/or escape neutralizing antibodies (26–28). The viral spike protein binding to the hACE2 protein is the first and crucial step in viral entry (29–34). The spike protein makes contact with hACE2 using 16 residues of the 223-amino-acid-long RBD forming multiple polar and hydrophobic interactions (35). The binding strength between RBD and hACE2 thus directly influences infection dynamics and, potentially, disease progression. Starr et al. (36) exhaustively assessed the impact of single–amino acid changes in the RBD of the SARS-CoV-2, quantifying the effect on RBD expression and hACE2 binding. It was revealed that most amino acid changes (i.e., 84.5%) are detrimental for RBD expression and hACE2 binding, around 7.5% of amino acid changes are neutral, but about 8% enhance hACE2 binding. The corresponding amino acid changes in RBD that lead to enhancements in binding with hACE2 can potentially become additive in their contribution to receptor affinity. Even though the RBD accounts for only 2% of the amino acid changes observed in the entire spike protein (37), it is the target for more than 90% of the neutralization antibodies generated by humoral response (38). Therefore, RBD is likely the most susceptible target to antigenic escape by amino acid changes. Consequently, amino acid changes in RBD that can increase binding affinity with hACE2 and/or adversely affect antibody neutralization have been extensively mapped by high-throughput mutational studies (39, 40). For example, the amino acid change Y453F in the RBD present in the ΔFVI (Danish mink) variant increases the binding affinity to hACE2 by fourfold (41) while also managing to partially evade the monoclonal antibody REGN10933 present in the Regeneron antibody mixture (42). These studies highlight the importance of monitoring single and multiple amino acid changes in the spike RBD and their potential for increased binding affinity with ACE2 and/or immune escape. It is important to note that, although the binding of the viral RBD with the hACE2 receptor is a necessary step, it is not sufficient to cause a productive viral infection. Proteolytic cleavage of S1/S2 and S2′ sites is also needed to expose the fusion peptide, enabling membrane fusion followed by viral entry at the surface or upon endocytosis (43). Furthermore, the host cellular environment must be permissive to viral RNA genome replication, translation to proteins, and assembly into new virions (44). Nevertheless, there is an unmistaken trend line in the accumulation of variants with amino acid changes that improve binding affinity of the viral RBD with hACE2. Upon assessing ∼1.5 million sequences deposited in the Covid-19 Mutation Tracker (CovMT) (45) database [which is based on data from the Global Initiative on Sharing Avian Influenza Data (GISAID) (46)], we found that approximately 1 million sequences had at least one single–amino acid change in the RBD. Of these, 92% (∼970,000 sequences out of 1 million) involved an amino acid change in the RBD that improves binding to hACE2 as measured by deep mutational scanning (36). This implies that binding-improving amino acid changes in the RBD are at least 11-fold enriched among circulating variants. This observation further underscores the importance of assessing variants with improved binding to hACE2 using prospective computational studies.

Computational methods can help assess the mechanistic role of the amino acid changes occurring in circulating viral variants and also predict potentially problematic amino acid changes that have not been identified so far. In a recent study, Chowdhury et al. (35) biophysically characterized the binding interactions of human ACE2 with SARS-CoV-2 and SARS-CoV, uncovering the molecular details associated with the increased infectivity of CoV-2, relative to CoV. In another effort, Mohammad et al. (47) calculated that the D614G variant has a higher computational binding interaction energy with furin. This was later experimentally corroborated, revealing that the D614G change both increases RBD accessibility to binding with hACE2 (11) and enhances the efficiency of furin cleavage (48). Zhou et al. (28) performed molecular dynamics (MD) simulations and molecular mechanics/Poisson−Boltzmann surface area analysis on the N439K variant suggesting a higher binding affinity to hACE2 and resistance to the antibody REGN10987. These findings were supported by experimental evidence for the N439K variant escaping multiple neutralizing antibodies, including REGN10987 (28). Several studies focus on testing the effect of one or several key single mutations, but systematic methods to predict and analyze a wider multimutational landscape are still lacking. It is worth noting that Chen et al. (49) used an algebraic topology-based machine learning (ML) model to quantify the binding free energy changes of RBD from several existing CoV-2 variants. However, the performance of the method used was tested on the general SKEMPI-2.0 (50) dataset, which is not SARS-CoV-2 specific. Recently, Laurini et al. (51) performed a computational mutagenesis of the RBD−ACE2 interface residues and assessed changes binding energies using MD simulations and validated using experimental data.

Several computational approaches have been developed to predict the effect of amino acid substitutions on protein−protein binding affinity. Some of them use energies directly from molecular mechanics−based empirical force fields such as FoldX (52) and Rosetta (53, 54) or energies from molecular mechanics–generalized Born surface area (MM-GBSA) analysis of ensembles obtained from MD simulations (55). Other methods, such as Single Amino Acid Mutation based change in Binding free Energy (SAAMBE) (56) and BindProfX (57), use a combination of biophysical energies and residue-level structural properties or sequence-based conservation profiles, respectively. Purely statistical potentials such as BeAtMuSiC (58) and Contact potentials (59) have also been explored. Updating the weights of energy terms using experimentally determined ΔΔ*G*_bind_ defined as the change in the free energy of binding upon amino acid changes (i.e., ΔΔ*G*_bind_ *=* Δ*G*_variant_ − Δ*G*_WT_) has been shown to improve the prediction performance of molecular mechanics−based force fields such as Rosetta (60, 61). The recently introduced ML−based method TopNetTree achieves a better correlation coefficient over several existing methods on two benchmark datasets, Antibody-Bind (AB-Bind) and Structural Kinetic and Energetic database of Mutant Protein Interactions (SKEMPI) (62). Despite the existence of many different ΔΔ*G*_bind_ prediction methods, as reviewed recently (63), performance is not always robust on unseen datasets not part of training data. The major limiting factor contributing to test set prediction inaccuracies is the paucity of experimental datasets (on ΔΔ*G*_bind_) with good coverage of both types and locations of amino acid changes (63).

**Fig. 1.**
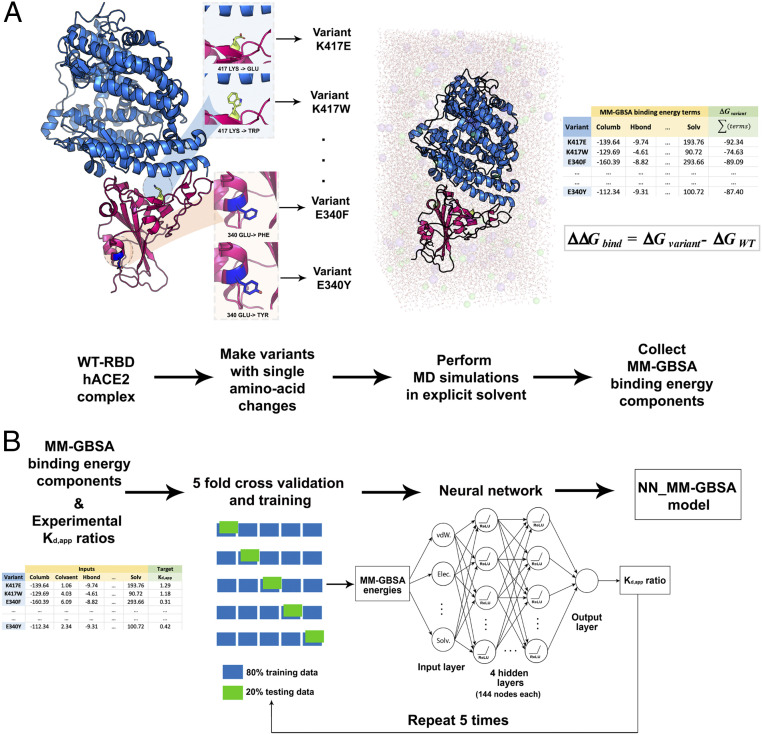
Schematic representation of the workflow for building NN_MM-GBSA model. (*A*) MD simulations are performed for each single-point amino acid substitution variant in explicit solvent followed by MM-GBSA analysis to calculate the decomposed components of binding energies. (*B*) MM-GBSA binding energy components are fed as inputs to the NN with the experimental *K*_D,app_ ratios as the regression target. The model is trained using five cycles of the fivefold cross-validation procedure.

In this work, we first tested the predictive power of both parameterized force fields [i.e., Rosetta (53, 54)] and detailed MM-GBSA (64–66) analysis of MD simulation trajectories with explicit water molecule treatment (67, 68) (Table 1) in reproducing experimental RBD−hACE2 binding affinity data reported by Starr et al. (36). Predictions for both provided only partial agreement with experimental data (i.e., *r* = 0.33 for MM-GBSA). Therefore, we next used experimental RBD−hACE2 binding energy terms to train a neural network (NN) regression model (NN_MM-GBSA) using the decomposed MM-GBSA energy terms as features and the experimental dissociation constants (*K*_D,app_) ratios between the variants and the wild-type as the regression target. Fig. 1 pictorially illustrates the computational pipeline employed to build the model. Agreement between experiment and the NN_MM-GBSA model predictions was significantly better than raw MM-GBSA energies, reaching a correlation coefficient of *r* = 0.73 and an accuracy of 82.8% for correctly classifying of the effect of amino acid changes as improving or worsening the binding affinity. The NN_MM-GBSA model also predicted the enhanced binding affinities of the RBD from currently circulating SARS-CoV-2 variants (Table 2). The achieved accuracy of prediction suggests that this model can be a useful tool for the computational assessment of both current and emerging SARS-CoV-2 variants. The source code for the NN_MM-GBSA model is available on GitHub at https://github.com/maranasgroup/NN_MM-GBSA_CoV2.

**Table 1.** Comparison of prediction performance of Rosetta and MM-GBSA with the regression models trained on Rosetta or MM-GBSA energies

Method	Correlation coefficient *r*	%VC
NN_ MM-GBSA	0.73 (0.03)	82.80 (1.98)
NN_Rosetta	0.56 (0.08)	74.03 (2.75)
Linear_regression_MM-GBSA	0.54 (0.17)	67.23 (1.20)
Rosetta	0.47	68.52
MM-GBSA	0.33	61.11

For the regression models, the SD obtained for the five repetitions of the fivefold cross-validation and training is shown in parentheses.

**Table 2.** Predictions of *K*_D,app_ ratios for amino acid changes found in circulating strains of SARS-CoV-2

Amino acid change(s)	SARS-CoV-2 variant lineage (102)	Experimental *K*_D,app_ ratio	NN_MM-GBSA *K*_D,app_ ratio
K417T	P.1(5) (γ)	0.55	0.65
K417N	B.1.351(4) (β)	0.35	0.61
L452Q	C.37 (9) (λ)	1.17	1.11
L452R	B.1.429(6), B.1.617.2 (8) (δ)	1.05	1.09
Y453F*	B.1.1.298 (23)	1.78	1.21
S477N	B.1.526(7)^†^	1.15	1.09
T478K	B.1.617.2 (δ)	1.05	1.11
E484K	B.1.351(4), P.1(5)(γ), B.1.1.7^†^(3)(α)	1.15	1.21
F490S	C.37 (λ)	1.00	1.10
S494P	B.1.1.7^†^(α)	1.00	1.04
N501Y*	B.1.351(β), B.1.1.7, P.1(γ)	1.74	1.22
E484Q	B.1.617(8)	1.07	1.09
E484K+N501Y	P.1, B.1.351	–	1.22
E484K+S477N	B.1.526^†^	–	1.10
E484Q+L452R	B.1.617	–	1.21
T478K+L452R	B.1.617.2(δ)	–	1.12
F490S+L452Q	C.37 (λ)	–	1.11
E484K+N501Y+K417T	P.1(γ)	–	1.22
E484K+N501Y+K417N	B.1.351(β)	–	1.22
E484K+N501Y+S494P	B.1.1.7^†^(α)	–	1.22

* Single–amino acid changes part of the training data.

^†^ Amino acid change detected in some sequences of lineage but not all.

## Results

### Dataset Preparation.

**Fig. 2.**
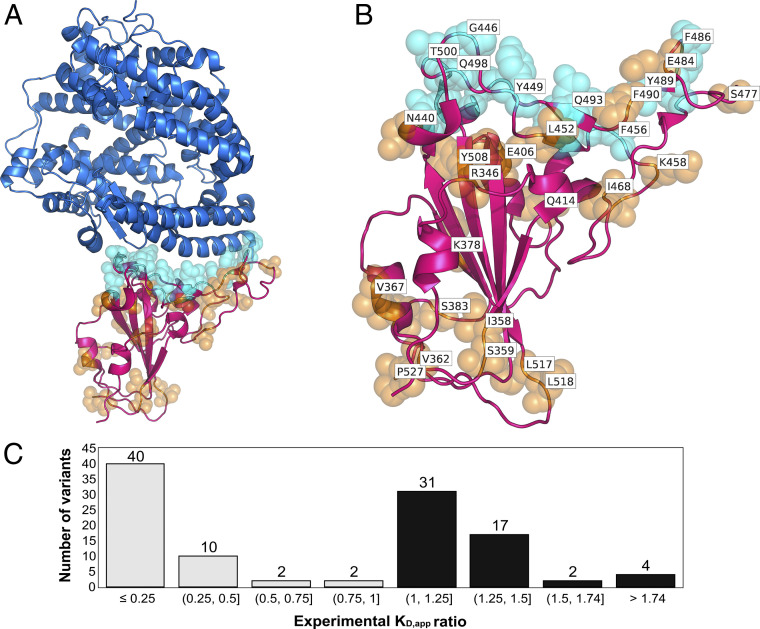
(*A*) The crystal structure of complex formed between RBD and hACE2. The ACE2 protein is shown as a cartoon representation in blue, and the RBD is shown in magenta. Residues of the RBD variants that are in direct contact with hACE2 are depicted as cyan spheres. Residues that are not in direct contact are orange. (*B*) Zoomed view of RBD in (*A*) with residues labeled explicitly. (*C*) Histogram showing experimental *Κ*_D,app_ ratios for all 108 RBD variants in the training dataset. The histogram bars in black denote the number of variants in the training set with increased binding affinity compared to WT (*Κ*_D,app_ ratio*>*1.0), and the bars in gray indicate the variant counts with decreased binding affinity (*Κ*_D,app_ ratio *<* 1.0).

The three-dimensional (3D) coordinates of the SARS-CoV-2 RBD in complex with human ACE2 were obtained from the Protein Data Bank (PDB) (69) entry 6LZG (70). There exist 20 RBD residues that directly make contact with hACE2 and make strong interactions at the binding interface (36). This gives 380 possible single–amino acid variants upon changing each one of the 20 RBD residues into the remaining 19 amino acids. Of these, we chose all 27 variants with an increased binding affinity and 54 variants with lower binding affinity compared to the WT. The dataset was balanced by adding another 27 variants that exhibited binding enhancement although not in direct contact with hACE2. These variants were selected to maintain roughly an equal number of positive (binding energy improving) and negative (binding energy decreasing) variants in the dataset. The 108 variants selected (Fig. 2*B*) (Dataset S1) formed the training dataset for this study and are used for the fivefold cross-validation training (see *Methods*). Another set of 54 variants (not a part of the training set of 108 variants) was selected as a blind test set that was not used in any of the training/validation procedures (Dataset S2).

All RBD variants in the dataset were computationally modeled using Rosetta (54, 55) and analyzed for changes in binding affinity with hACE2 compared to the WT RBD. Experimental data on variant binding affinities were obtained from the deep mutagenesis study by Starr et al. (36). The study reported apparent dissociation constant *K*_D,app_ ratios for all possible variants with single–amino acid changes at every RBD position. A *K*_D,app_ ratio (i.e., *K*_D,app,variant_/*K*_D,app,WT_) for a variant greater than one implies stronger binding compared to WT, whereas a value less than one implies weaker binding (Fig. 2*C*). *K*_D,app_ ratios can be related to changes in the free energy of binding (i.e., ΔΔ*G*_bind_) as (*K*_D,app*,*variant_)/(*K*_D,app*,*WT_) = exp(−ΔΔ*G*_bind_/RT). This enables direct comparison of experimental measurements with estimates of changes in binding energies from MM-GBSA and other computational methods (see *Methods* for details).

### Binding Affinity Change Prediction for Variants Using MM-GBSA Values from MD Simulations.

For each RBD variant, we first performed MD simulation of the hACE2−RBD complex followed by MM-GBSA analysis on frames derived from the simulation to calculate binding energies. For each variant, 48 independent initial configurations of the complex were generated by Monte Carlo minimization (53, 71) (see *Methods*). Starting from each configuration, a 4-ns unconstrained MD simulation was carried out which, together, sum up to a 192-ns-long trajectory for each variant. We used a sequence of short simulations (72), starting from several independent configurations instead of one long simulation trajectory, as it led to faster convergence. The 3D coordinates of the complex were extracted from each trajectory upon removing the solvent molecules after every 0.1 ns, generating 1,920 different frames for each variant. The simulations were found to be equilibrated at the interface, as indicated by the SD in RMSD ranging between 0.45 and 0.61 Å for all 108 variants (*SI Appendix*, Figs. S1 and S2). MM-GBSA energies were calculated for all frames (see *Methods*) and subsequently averaged in 60 bins chosen randomly, to obtain an ensemble of 32 binding energy predictions for each variant. The mean value of the ensemble of 32 predictions was chosen as the predicted binding energy Δ*G*_variant_ of the variant. The binding energy change for each variant ΔΔ*G*_bind_ was then obtained by subtracting the binding energy of the WT RBD−hACE2 complex Δ*G*_WT_. A negative ΔΔ*G*_bind_ value (corresponds to *K*_D,app_ ratio > 1) indicates improved binding affinity with hACE2, whereas a positive ΔΔ*G*_bind_ value (corresponding to *K*_D,app_ ratio < 1) implies lowered binding affinity.

**Fig. 3.**
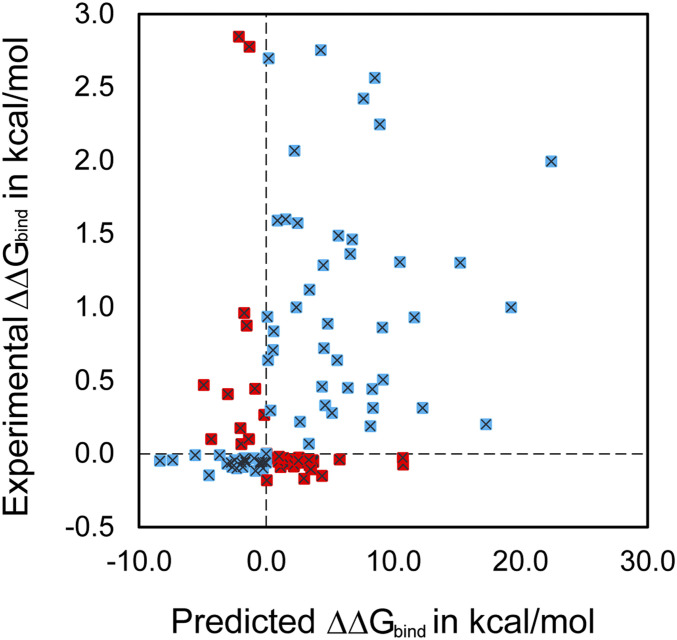
ΔΔ*G*_bind_ prediction performance of MM-GBSA binding energies on 108 RBD variants. Dashed horizontal and vertical lines are drawn for reference at experimental and predicted ΔΔ*G*_bind_ = 0. Shown in blue are variants for which the effect on binding affinity (sign of ΔΔ*G*_bind_) is predicted correctly compared to the experimental value. Those predicted incorrectly are shown in red.

Using this computational workflow, we calculated the ΔΔ*G*_bind_ for the balanced dataset of 108 RBD variants. Note that a balanced training set was maintained, to alleviate the risk of biased predictions due to having more variants with worsening or improving binding affinities. We scored classification predictions using the percent recovery of correct variant classification (%VC) in terms of the direction of change in the binding affinity compared to WT. The quantitative binding affinity prediction was scored using the Pearson correlation coefficient *r* (see *Methods*) between predicted and experimental ΔΔ*G*_bind_ values. We found that (Table 1) Rosetta slightly outperforms MM-GBSA in both prediction of the direction of change (i.e., %VC) and *r* value. This could be because of the poor scaling of the respective energy terms in MM-GBSA for the experimental system, leading to some outliers having very large predicted values (Fig. 3) and a lower *r* value (i.e., *r* = 0.33) than Rosetta (i.e., 0.47). In addition to the energy function from Rosetta (54), three other computational servers were tested for the prediction: mCSM-PPI2 (73) utilizing graph-based signatures, the random forest model MutaBind2 (74) trained with molecular mechanics energies (75) and evolutionary scores (76), and SAAMBE-3d (77, 78) which uses an ML model trained on structural features. Using MutaBind2 and mCSM-PPI2, the performance in both %VC and *r* value was worse than that of both MM-GBSA and Rosetta. The predictions from SAAMBE-3d led to a good correlation value *r* but were very poor in %VC (=53%), almost the same as random prediction. This may be due to the fact that Rosetta and MM-GBSA attain a higher fidelity in the description of the underlying biophysics by using a detailed fully atomistic description of interactions and hence are better at distinguishing improving vs. worsening variants. Note that, because the numerical values ΔΔ*G*_bind_ for variants improving the binding affinity are quite small (maximum of ∼ −0.3 kcal/mol) compared to those worsening the binding affinity (maximum of ∼ +2.5kcal/mol), both metrics %VC and *r* need to be simultaneously high to indicate robust prediction. Nevertheless, prediction metrics %VC and *r* calculated for MM-GBSA (or Rosetta) did not attain values that reflect reliable quantitative prediction. We thus focused on improving prediction fidelity by attempting to capture nonadditive contributions of the respective energy terms. This was accomplished by not merely using various energy terms in an additive fashion to assemble the overall binding energy but, instead, by relying on an NN to construct a nonlinear reassortment of these energy terms.

### NN Regression Model Trained on MM-GBSA Energies and Experimental *K*_d,app_ Ratios.

An NN regression model with a single input and a single output layer was built, targeting quantitative prediction of the *K*_D,app_ values for the 108 RBD variants. The MM-GBSA ensemble of energies obtained from the MD trajectories of each variant was fed as input features to the NN. The input layer had 18 nodes for feeding in the 18 MM-GBSA energy terms (see *Methods* for description of all terms). The output from the input layers passed through each of the four fully connected hidden layers with 54 nodes in each layer (see *Methods* for details on how the NN parameters were obtained by optimization). After passing through the hidden and output layers, a single predicted value for *K*_D,app_ was generated. The model was trained to minimize the mean sum of squared error between the predicted *K*_D,app_ and experimental *K*_D,app_ values (see *Methods* for details). When making a prediction, each of the 32 sets of energies was fed into the trained model to get a single *K*_D,app_ prediction, and the final prediction value was recovered as the mean of predictions from all 32 ensembles. The overall computational workflow is summarized in Fig. 1.

**Fig. 4.**
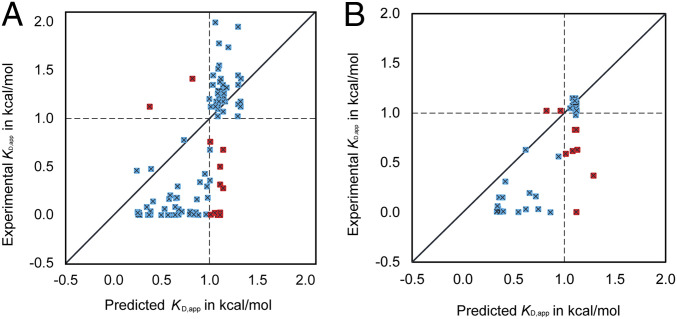
(*A*) *K*_D,app_ ratios predicted by NN_MM-GBSA vs. experimental ratios obtained using five cycles of fivefold cross-validation study on the Training & Validation dataset of 108 RBD variants. (*B*) Blind test dataset of 54 RBD variants. An average correlation coefficient of *r* = 0.73 (SD = 0.03) and average %VC of 82.80% (SD = 0.9802) were achieved for the validation set, and *r* = 0.79 (SD = 0.03) and average %VC of 82.8% (SD = 2.01) were achieved for the blind test set. The mean-squared error was 0.28 (SD = 0.04) and 0.20 (SD = 0.02) for validation and blind test sets, respectively. The solid diagonal line *y* = *x* and the dashed horizontal and vertical lines at experimental and predicted *K*_D,app_ ratio = 1 are drawn for reference. Shown in blue are variants that were correctly classified as improving (or worsening) binding affinity with hACE2, and, in red, are the ones that were misclassified.

During training and assessment of the model, a fivefold cross-validation procedure was followed. In each cross-validation cycle, the 108 variants are randomly assigned to five groups of approximately equal size. Four of these subsets were used as the training sets, whereas the fifth became the testing set. This approach was chosen so that the testing set used to assess the prediction performance of the NN model is never used to train the predicting NN model. This fivefold cross-validation was repeated 10 times using random reassignments for the testing set. This led to the construction of 5 × 10 = 50 independently trained NN models which had an average value of *r* = 0.73 (obtained across the 50 models) and an SD of only 0.03, implying both robust and accurate prediction (Fig. 4*A*). Notably, the correlation coefficient of prediction improved by more than twofold compared to the MM-GBSA method (i.e., *r* = 0.33), indicating that a higher-order nonlinear structure, relevant to ΔΔ*G*_bind_ prediction embedded in the energy terms, was captured by the NN model. Correct variant classification (i.e., %VC) was also improved from 64 to 82.8% (Table 1). We also evaluated the performance of the NN on the blind test set of 54 variants using NN_MM-GBSA trained on the entire dataset of 108 variants (Fig. 4*B*). The performance achieved on the blind test had an *r* value of 0.79 and %VC of 80.41, very close to those obtained on the validation data. This blind test analysis alludes to the robustness of NN_MM-GBSA on unseen data and indicates that it is not prone to overfitting.

As a methodological check, we also explored whether the nonlinear nature of the NN model is needed to reach the gains in prediction or whether a linear regression model could reweight the energy terms in a linear fashion and achieve similar performance. We found that a linear regression model only improved the correlation coefficient *r* from 0.33 to 0.54 and %VC from 61.11 to 67.23% in comparison with the MM-GBSA prediction method. This implies that the higher-order nonlinear reassortment of energy terms is required for reaching improved prediction fidelity. As a follow-up, we also explored whether the energy terms from Rosetta (53, 54) could be used instead of the ones from MM-GBSA to construct an NN model of equivalent predictive ability. We found that the gains in *r* and %VC for an NN model trained on the Rosetta energy terms were less than those seen when trained on MM-GBSA energies (i.e., *r* = 0.57, %VC = 74.33). This may be because the explicit water treatment embedded in MD simulations is essential for correctly describing water-mediated hydrogen bonding and other electrostatic contacts at the interface while also enabling the sampling of a larger conformational landscape necessary for capturing binding affinity changes due to nonlocal structural changes (72). Note that the Rosetta energy function captures solvation effects implicitly without an explicit treatment of water molecules.

As a further demonstration that the NN_MM-GBSA model captures variant-specific information and does not simply carry out numerical fitting, we performed a data scrambling test. Specifically, we reassigned the variant definition (i.e., corresponding amino acid change) to randomly chosen input energy terms, thereby destroying any variant-specific correspondence with the input features. We gradually increased the fraction of data scrambled and reevaluated NN_MM-GBSA model performance. We found that, as the fraction of scrambled data increased, the performance of the NN_MM-GBSA model declined (*SI Appendix*, Table S4). The %VC dropped from the original 82.8% to 50.37% (almost entirely random). This test reaffirmed that the NN_MM-GBSA model indeed captured variant-specific information.

### NN_MM-GBSA Model Prediction of *K*_D,app_ Ratios of Circulating SARS-CoV-2 Strains.

**Fig. 5.**
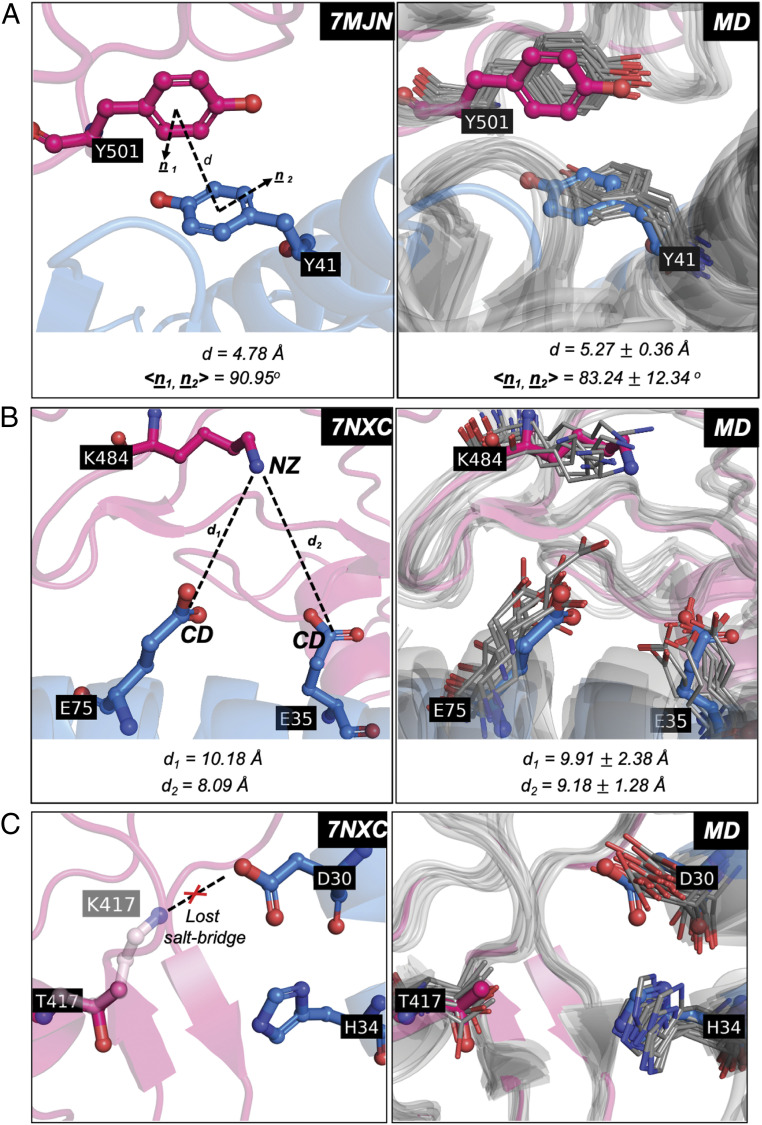
Local environments altered by the amino acid changes N501Y, E484K, and K417T as seen in MD simulations are compared with the cryo-EM structures 7MJN of the N501Y variant and 7NXC of the P.1 variant. The RBD is shown in magenta and the hACE2 is shown in blue cartoon representation. Amino acid side chains from the cryo-EM structures are shown in stick representation, and the corresponding side chains from the MD simulations are shown in line representation. (*A*) Comparison of a pi−pi interaction formed in the variant N501Y as seen in cryo-EM structure 7MJN vs. as seen in a representative set of 10 MD snapshots. The pi−pi interaction is characterized by the distance *d* between geometric centers of the two tyrosine residues and the angle <n_1_, n_2_> between their normal vectors. For MD snapshots, average and SD of *d* and <n_1_, n_2_> computed across 192 ns simulation are shown. (*B*) Comparison of the proximity of lysine in E484K variant to glutamate residues at 35 and 75 of hACE2 as seen in cryo-EM structure 7NXC vs. as seen in a representative set of 10 MD snapshots. The distances *d*_1_ and *d*_2_ for 7NXC are shown, and those shown for MD snapshots are average and SD computed across 192-ns simulation. (*C*) Figure of the close-by residues of threonine in the variant K417T as seen in cryo-EM structure 7NXC, indicating the loss of salt bridge from WT (lysine residue of the WT taken from 6LZG crystal structure is shown as transparent stick). The corresponding 10 representative MD snapshots are shown to the right in comparison.

Several amino acid changes have been identified in the spike protein of several of the currently circulating SARS-CoV-2 variants (20). Of the underlying single–amino acid changes, some were part of our balanced training set (i.e., N501Y, L452Q, Y453F), whereas others (i.e., K417T, K417N, E484K, S477N, L452R, T478K, F490S, S494P) were not. The predicted *K*_D,app_ ratios along with experimental values (when available) and lineage names of variants that contain the corresponding amino acid changes are tabulated in Table 2. In all cases, amino acid changes were correctly classified as improving or worsening (i.e., %VC = 100) with a good quantitative agreement (Table 2). Notably, variant B.1.351 has the amino acid change N501Y first seen in B.1.1.7 along with additional changes E484K and K417N in the spike (79, 80). The E484K change has been shown to be responsible for evasion of neutralization by several antibodies (78, 81), whereas the N501Y change has been associated with increased binding affinity to hACE2 (36) and increased transmission (82). Our MD simulation results suggest that the Y501 residue of the RBD in the variant N501Y forms a new electrostatic interaction with residue Y41 of hACE2 through a T-shaped pi−pi stacking interaction (Fig. 5*A*), as also validated by the recently published cryoelectron microscopy (cryo-EM) structure (79) N501Y-RBD in complex with hACE2 (Fig. 5*A*). Also, the amino acid change E484K places a lysine residue close to residues E75 and E35 of hACE2, causing more favorable electrostatic interactions compared to the WT (Fig. 5*B*). This led to the computational prediction of the formation of a weak salt bridge (although only seen in 4% of frames during MD simulations) contributing to its improved binding affinity (Table 2). Also, the distances between the nitrogen atom of side chain of K484 from the carbonyl atoms of side chains of E75 and E35 as sampled by the MD simulations closely agree with those from the cryo-EM structure (83) of P.1 variant (Fig. 5*B*). In contrast, amino acid change K417T leads to the loss of a salt bridge (Fig. 5*C*), presumably causing the observed decrease in binding affinity as also predicted by NN_MM-GBSA (Table 2). We further assessed the accuracy of structure recovery of the P.1 (γ) variant by our MD simulations by comparing it with the cryo-EM structure 7NXC (83). The MD snapshots yielded an average all-atom RMSD of 0.88 Å of the interface residues and an average RMSD of 1.57 Å of the entire complex when superimposed on the cryo-EM structure (*SI Appendix*, Tables S1 and S2). Good agreement in recovering backbone configurations was also demonstrated by the MD simulations of P.1 variant (*SI Appendix*, Fig. S3). Table 2 also includes predictions for multiple simultaneous amino acid changes present in some circulating variants. Alas, experimental values are not available to this date, which prevents any direct comparison. Nevertheless, significantly higher binding affinities were predicted for all of the double and triple amino acid variants tested and present in currently circulating isolates (Table 2).

## Discussion

The NN_MM-GBSA model was developed by a two-step procedure that uses binding energy terms calculated for SARS-CoV-2 RBD variants from MM-GBSA to train an NN to reproduce corresponding experimental values (36) of binding affinity changes of the RBD variants with hACE2. The model predicts both qualitative and quantitative effects of amino acid changes in the RBD of the spike protein on changes in binding affinity with hACE2. Using a balanced training set of 108 variants, the model achieved a Pearson correlation coefficient of 0.73 between predicted and experimental values for the *K*_D,app_ ratios. In addition, the recovery of the correct effect of an amino acid change (i.e., improving or worsening binding) was 82.8%. We also found the prediction to be quite robust in performance on a blind test set of 54 variants, achieving an *r* value of 0.79 and %VC of 80.41. Notably, Starr et al. (36) exhaustively assessed a total of ∼4,000 RBD variants for their binding affinity changes with hACE2, whereas, in this study, we used only a small fraction of the dataset (108 variants). Furthermore, as we continued to add additional members to the training dataset of 108 variants, no clear trend line was observed indicating any systematic change in model performance.

The true value of NN_MM-GBSA is not the assessment of variants with single–amino acid changes, but the surveillance of multiple–amino acid change variants. We predicted the change in binding affinity upon the amino acid changes E484K+N501Y+K417N present in B.1.351(β) and E484K+N501Y+K417T present in P.1(γ). We found that NN_MM-GBSA predicts a significantly increased affinity for hACE2 (Table 2) for both. This suggests that the effect of amino acid changes E484K and N501Y dominates the effect of K417N or K417T, which are both known to decrease the binding affinity by themselves, and this is possibly the reason for the convergent association of these amino acid changes seen in the lineages B1.351(β) and P.1(γ) (80). Furthermore, the amino acid changes T478K+L452R and F490S+L452Q that emerged in the B.1.617.2 (δ) and C.37 (λ) lineages first seen in India (8) and in Peru (9), respectively, were predicted to have an improving binding affinity to hACE2 by NN_MM-GBSA (Table 2).

Importantly, the structural recapitulation of these variants by MD simulations was found to be in good agreement with the cryo-EM structures 7NXC (83) and 7MJN (79). Hence, using relatively 48 short (4 ns) trajectories, each starting from a different backbone conformation generated using Rosetta, proved to be sufficient for sampling crucial structural features of RBD variants. This could be because the protein backbone conformations in RBD variants were not perturbed significantly (*SI Appendix*, Table S1) compared to the RBD structure of WT virus. Note that, previously, a similar combination of Rosetta and short MD simulations has been used to achieve accurate structure refinement by iterative sampling (84).

A drawback of NN_MM-GBSA is that it requires a priori MD simulation of the variant under evaluation and collection of all energy terms using MM-GBSA analysis. This is computationally costly, as a single calculation requires, on average, a total of ∼72 GPU-hours on an Nvidia Tesla P100 and 24 CPU-hours on Intel Xeon 2.8GHz processors. Ideally, one could simply use existing energy terms generated from the balanced training set of 108 variants to make predictions for novel variants. However, this would require training an NN model with more than just energy terms as descriptors. The use of sequence and/or structural features could provide a tractable path forward in this direction.

To assess the potential impact of glycosylated sites on RBD and hACE2 on the binding affinity, we repeated the MD simulation with the presence of glycan residues at position 343 in the spike and positions 53, 90, and 322 in ACE2 starting from the crystal structure 6LZG. We found insignificant changes in the binding affinity for the WT complex (*SI Appendix*, Table S5). While a recent experimental study suggested that there is only a subtle influence of hACE2 glycosylation on its binding strength to RBD (85), other efforts (86) suggested a more significant contribution of glycosylation to binding. This implies that carefully tailored studies are still needed to quantify the effect of glycans on changes in binding affinity upon amino acid changes.

In principle, NN_MM-GBSA can also be used to assess the potential of SARS-CoV-2 to infect and adapt to other nonhuman hosts, by assessing the binding energies of the spike RBD with the animal ACE2 receptors. However, the structures of nonhuman ACE2 are currently unavailable [except for bats (87) and felines (88)]; therefore, the first step would require modeling the 3D structures of ACE2 receptor and ACE2−spike complexes for the examined species. Several efforts along this direction have been carried out for livestock and companion animals (35, 89), and accurate assessments for high-risk animals are urgently needed, since several animal species are proving to be susceptible by natural infection [gorillas (90), otters (91)] or experimental infection [deer (92), cattle (93), pigs (94)]. Assuming that training of NN_MM-GBSA using hACE2 data is robust, it could, in principle, be used to assess the relative affinity of the RBD of circulating variants for various animal ACE2s prospectively. Crucially, our methodology can detect problematic amino acid changes and assess the potential of increased cross-species transmission for circulating (or predicted) variants.

## Methods

### Rosetta Calculations for Independent Structure Generation.

The 3D coordinates of SARS-CoV-2 viral spike RBD in complex with hACE2 were extracted from the crystal structure with PDB entry 6LZG (70). The obtained WT model was first preprocessed by removing all solvent molecules and all non−amino acid residues. Then, for each of the 108 RBD variants with single-point amino acid changes, 3D coordinates were generated using RosettaScripts (95). First, the PackRotamers mover was used to build the variants with amino acid changes and repack the rotamers. Then, for each variant, 48 independent configurations for MD simulations were generated using the Relax (71) energy minimization protocol.

### MD Simulations and MM-GBSA Analysis.

For each variant, the 3D coordinates of 48 independent configurations obtained using Rosetta (as described above) were prepared using the protein preparation wizard (96) protocol of Maestro in Schrödinger suite (v2019.4). Each configuration was then solvated with water using the tip3p (68) model in an orthorhombic box with 10-Å buffer distance in each dimension. The residual charges were neutralized by adding Na^+^ and Cl^−^ ions at a salt concentration of 0.15 M. The solvated systems were minimized and preequilibrated using the default relaxation protocol of Desmond (97) followed by a 4-ns production run using the amber99sb-ildn (67) force field at 300 K and 1 atm. The simulations were perfromed in isothermal-isobaric ensemble (NPT) ensemble with periodic boundary conditions using particle mesh Ewald (98) for long-range interactions. A time step of 2.0 fs was used, and a cutoff distance of 9.0 Å was chosen for nonbonded interactions.

For each variant, the 4-ns trajectory for each of the 48 configurations was sampled at an interval of 0.1 ns, generating 1,920 snapshots in total. For each snapshot, the Prime/MM-GBSA analysis (66) was performed using thermal_mmgbsa.py script from the Schrödinger suite. The MM-GBSA analysis produces the binding energy and its constituent eight individual energy terms, that is, Coulombic, covalent, van der Waals, lipophilic, generalized Born electrostatic solvation, hydrogen bonding, π−π packing, and self-contact correction terms. Another set of values for these nine terms have also been calculated by not accounting for receptor and ligand conformational changes needed to form the complex. Due to a high degree of variation in the energies, we averaged data from 60 snapshots to produce a single set of averaged energy terms in the dataset. Thus, a total of 1,920 snapshots generate 32 sets of averaged energy terms for each variant. In total, these 18 energy values were utilized as the input features for NN construction.

### NN for MM-GBSA Energies (NN_MM-GBSA).

#### Dataset generation.

MM-GBSA analysis was used to generate 18 energy components (as described above) fed as the input features for the NN_MM-GBSA model. Each input energy term across the whole dataset was scaled independently to have zero mean and a variance of one. The output target was set to the experimental apparent dissociation constant *K*_D,app_ ratios, (*K*_D,app_)_variant_/(*K*_D,app_)_WT_. The experimental data for the 108 RBD variants (Dataset S1) were obtained from Starr et al. (36).

#### Model architecture.

The NN has a single input layer, a single output layer, and four fully connected hidden layers with 54 nodes per layer, forming 18−(54−54−54−54)−1 structure. The rectified linear unit was used as the activation function for all the hidden layers, and the dropout regularization method was applied to hidden layers, with a dropout rate of 0.5 for the first and last hidden layers, and 0.75 for the rest.

#### Hyperparameter optimization.

We conducted the Bayesian hyperparameter optimization (99) as implemented in the Hyperopt package (hyperopt.github.io/hyperopt) to choose the following five hyperparameters: number of snapshots to average over (between 60 and 480), number of hidden layers beside the input and output layers (between 1 and 4), number of neurons per layer per input element (between 2 and 8), learning rate (between 0.001 and 0.01), and Adam optimizer weight decay parameter (between 0.0001 and 0.01). The loss function to minimize was defined as follows:

$$\text{loss}=\left(\%{\text{VC}}_{\text{training}}-{\text{\%VC}}_{\text{validation}}\right)/15+\left({\text{MSE}}_{\text{validation}}-{\text{MSE}}_{\text{training}}\right)/0.2,$$

where %VC is the correct variant classification percentage, and MSE is the mean-squared error. The loss function represents the difference between the training and validation sets. The constants 20 and 0.15 were chosen to roughly scale %VC and MSE values to similar orders of magnitude. Three individual fivefold cross-validations were performed, results were averaged to evaluate the loss function in each round, and a total of 50 iterations of optimization were performed to achieve the final set of hyperparameters: number of MD snapshots to average over = 60, number of hidden layers beside the input and output layers = 4, number of neurons per layer per input element = 3, learning rate = 0.003403, and Adam optimizer weight decay parameter = 0.0001236.

#### Model training.

The model was trained through back-propagation to minimize the mean-squared error between predicted *K*_D,app_ and target *K*_D,app_ values. Adam optimizer (100) was used to perform the back-propagation, with a learning rate of 0.003403 and weight decay of 0.0001236. The training was performed for 2,000 epochs, including the entire training data in each batch.

#### Model evaluation.

The 108 variants are used for model training using fivefold cross-validation. The evaluation consists of splitting the 108-variant database into five subsets. In one complete evaluation cycle, each of the five subsets was used as a validation set once, the rest constituted the training set, and a total of five such cycles were performed. We constructed a blind test set of 54 variants by randomly picking 27 variants from the categories of improving and worsening binding affinity. Note that, to make complete use of the data for predictions on variants listed in Table 2 and the blind test set, we used a model trained on the entire set of 108 variants.

Two metrics were employed to quantify the performance of NN_MM-GBSA model predictions: % correct variant classification (%VC), and the Pearson correlation coefficient (*r*). The %VC is the percentage of instances in which a variant is classified correctly as increasing or decreasing the binding affinity compared to WT. The Pearson’s correlation coefficient is defined as

$$r=\frac{\sum \left(x_{i}-\bar{x}\right)\left(y_{i}-\bar{y}\right)}{\sqrt{\sum {\left(x_{i}-\bar{x}\right)}^{2}\sum {\left(y_{i}-\bar{y}\right)}^{2}}},$$

where *x*_*i*_ and *y*_*i*_ are the target and prediction for the *i*th sample, and $\bar{x}$ and $\bar{y}$ are the mean value for all *x*_*i*_ targets and *y*_*i*_ predictions.

#### Model predictions.

The NN_MM-GBSA model predictions are based on a single model trained using 100% of the training data. When making the prediction for a variant, the ensemble of 32 sets of MM-GBSA energies are collected, and each set is used to make a single prediction for *K*_D,app_ using the model. The mean of 32 predictions is the final predictor of the *K*_D,app_ of the variant.

#### Implementation.

All codes were developed in Python using the PyTorch library.

#### Rosetta calculations for ΔΔG_bind_ prediction.

The complexes for 108 RBD variants were subject to Relax (71), with harmonic constraints to prevent the structure from deviating significantly from the crystal structure. During Relax, rotamers of amino acid residues within 8 Å of the mutated amino acid were only allowed to repack (local packing). All default parameters were used for Relax with the ref2015 energy function (54). At the end of Relax*,* a gradient minimization was performed using the lbfgs_armijo algorithm for 2,000 steps, after which the relevant metrics of binding were calculated using InterfaceAnalyzer (101). The binding energy, Δ*G*_variant_, of each variant was calculated as the average of dG_separated scores obtained from 30 independent Relax simulations. For each variant, a WT binding energy, Δ*G*_WT_, was calculated using the same protocol, by making a dummy amino acid change (change amino acid to itself). Finally, the change in binding energy ΔΔ*G*_bind_ was calculated as ΔΔ*G*_bind_ = Δ*G*_variant_ − Δ*G*_WT_.

## Data Availability

All relevant data pertaining to the results discussed in the paper are available either in the main text and *SI Appendix*. Representative raw MD trajectories can be made available upon request. Relevant simulation codes for generating the computational models have been deposited in the GitHub repository (https://github.com/maranasgroup/NN_MM-GBSA_CoV2).
